# Endovascular treatment of perigraft seroma in patient with prior hybrid thoracoabdominal repair using visceral bypass to relieve duodenal obstruction

**DOI:** 10.1016/j.jvscit.2022.07.016

**Published:** 2022-08-08

**Authors:** Tsutomu Doita, Kazuo Shimamura, Takayuki Shijo, Ryota Matsumoto, Shigeru Miyagawa

**Affiliations:** a Department of Cardiovascular Surgery, Osaka University Graduate School of Medicine, Osaka, Japan; b Department of Minimally Invasive Cardiovascular Medicine, Osaka University Graduate School of Medicine, Osaka, Japan

**Keywords:** Endovascular repair, Perigraft seroma, Visceral debranching bypass surgery

## Abstract

Perigraft seroma (PS) is a postoperative complication occurring after prosthesis placement. A 48-year-old man who had previously undergone visceral debranching bypass surgery as a part of hybrid thoracoabdominal aortic repair was referred to our hospital because of vomiting. Contrast-enhanced computed tomography revealed a duodenal obstruction resulting from compression by a PS located around the bypass graft and extending to the right renal artery. Endovascular relining of the visceral bypass graft using a covered stent was performed, resulting in immediate resolution of the duodenal obstruction and shrinkage of the PS. Endovascular repair can be considered as an effective option for treating a PS.

Perigraft seroma (PS) is a rare postoperative complication occurring after vascular prosthesis placement, with an estimated incidence of 1.2% to 1.3% following open aortic reconstruction.^1^ The most effective treatment has been reported to be graft replacement and resection of the seroma,^2^ although such redo surgery will often be highly invasive.^1^ In the present report, we have described a rare case of duodenal obstruction caused by PS development after visceral bypass surgery for a thoracoabdominal aneurysm that was successfully treated with an endovascular procedure.

## Case report

**Fig 1:**
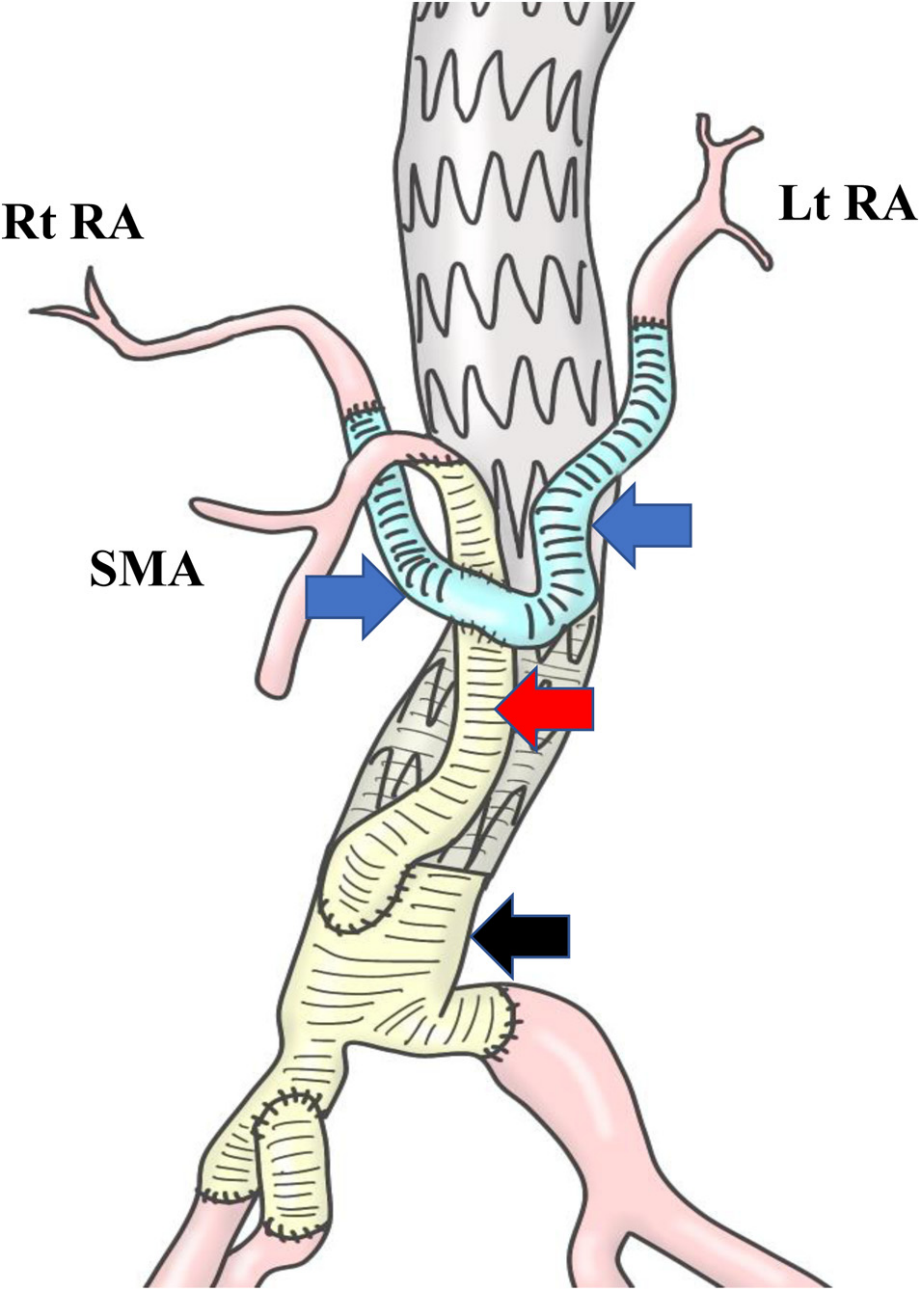
Visceral bypass surgery procedure. Cross-shaped bypass grafts were created with 12-mm woven (*red arrow*) and 8-mm expanded polytetrafluorethylene (ePTFE; *blue arrow*) grafts. First, the abdominal aorta and bilateral iliac arteries were replaced with woven grafts (*black arrow*). Next, the bilateral renal arteries (RAs) and superior mesenteric artery (*SMA*) were reconstructed using cross-shaped bypass grafts, which were finally attached to the replaced abdominal aorta woven graft. *Lt,* Left; *Rt,* right.

A 48-year-old man with Marfan syndrome had been referred to our hospital because of vomiting. He had a history of multiple aortic surgical procedures, including aortic root and total arch replacement for acute type A aortic dissection 13 years before and hybrid endovascular repair 8 years earlier for a residual thoracoabdominal dissecting aneurysm. During the latter operation, bilateral renal arteries (RAs) and the superior mesenteric artery were translocated using cross-shaped bypass, 12-mm woven and 8-mm expanded polytetrafluoroethylene (ePTFE), grafts, which we have previously reported^3^ (Fig 1). His physical examination at the latest follow-up visit showed a normal body temperature of 36.5°C, and abdominal palpitation indicated a soft flat abdomen. The blood tests revealed a white blood cell count of 7780/μL and C-reactive protein level of 1.39 mg/dL.

**Fig 2:**
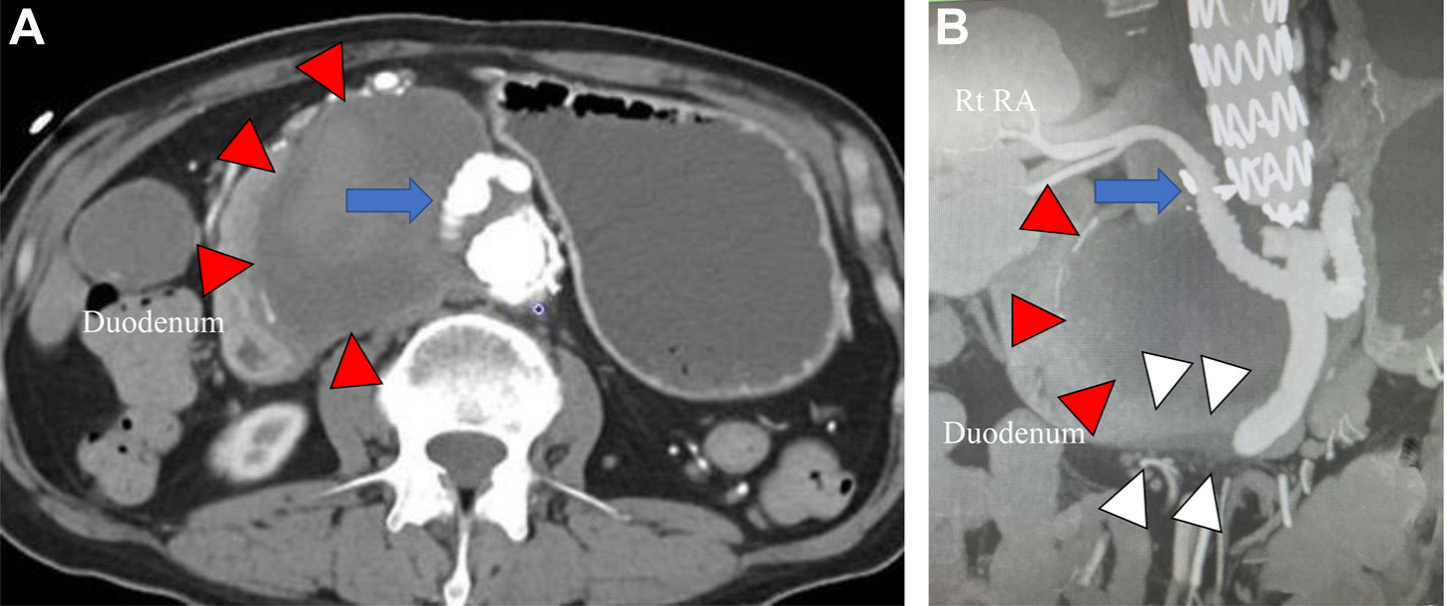
Contrast enhanced computed tomography (CT) images. **A,** Axial view. **B,** Coronal view. Contrast-enhanced CT revealed dilated regions in the stomach and duodenum and an abdominal low density mass (*red arrow*) compressing the third position (*white arrow*). The mass was ∼95 mm in size and located around the bypass graft to the right (*Rt*) renal artery (RA; *blue arrow*). No extravasation of contrast media was noted in the area of the mass.

A homogeneous low density mass in the abdomen 95 × 60 mm in size with a radiodensity of 42 Hounsfield units and causing compression of the third portion of the duodenum was shown by contrast-enhanced computed tomography (CT), with dilation of the first and second portion and stomach also noted (Fig 2). The mass was located around the visceral bypass graft to the right RA, with no extravasation of contrast media observed. Moreover, the CT findings did not indicate graft infection, perigraft gas formation, or a soft tissue abnormality, such as an ill-defined inflammatory mass or abnormally increased fat attenuation (Fig 2). An upper gastrointestinal endoscopy examination found no abnormal gastric mucosal lesion suggestive of an aortoenteric fistula. CT-guided drainage of the mass was performed, and culture testing confirmed no growth of bacterial organisms. Therefore, the suspected primary diagnosis was PS. Considering the location next to the right RA graft and the ePTFE graft material, it was speculated that a seroma had developed from the graft. Because of the high invasiveness of redo surgery with repeat laparotomy and repeat visceral bypass grafting, primary endovascular repair was planned. After confirming that cultures of the mass had remained negative for 6 days, the procedure was performed.

**Fig 3:**
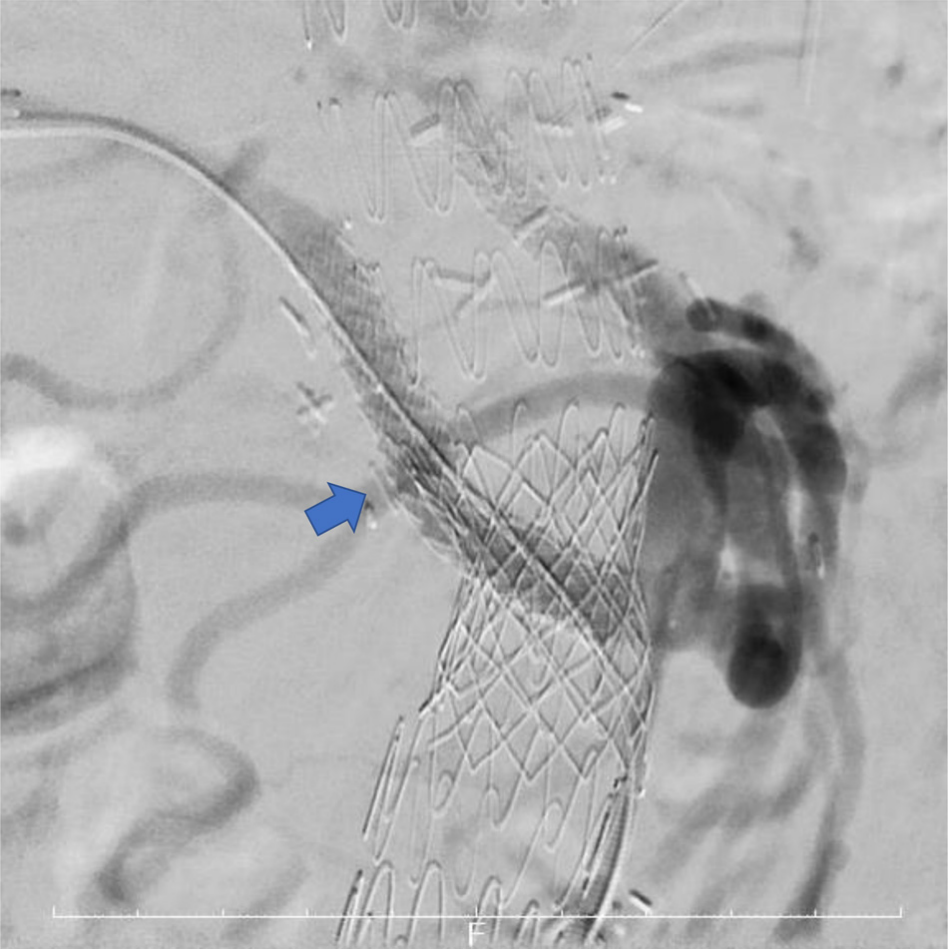
Angiography after placement of a VBX balloon expandable endoprosthesis (W.L. Gore & Associates). A VBX stent graft (*blue arrow*) was used to cover the end-to-end anastomotic part of the right renal artery (RA) through the crossed portion of the 8-mm expanded polytetrafluorethylene (ePTFE) and 12-mm woven grafts.

With the patient under general anesthesia and using transfemoral access, selective catheterization of the bypassed graft to the right RA was performed with an Impress 5F headhunter catheter (Merit Medical Endotek, South Jordan, UT). Next, an 8F, 45-cm Destination Guiding Sheath (Terumo Corp, Tokyo, Japan) was advanced over a 0.035-in. Rosen Wire Guide (Cook Medical Inc, Bloomington, IN) into the bypass graft to the right RA, with the device selected according to the caliber and length needed. Next, an 8 × 59-mm Viabahn VBX balloon expandable endoprosthesis (W.L. Gore & Associates, Flagstaff, AZ) was deployed in the ePTFE bypass graft to the right RA, which covered the end-to-end anastomotic part of the right RA through the crossed part of the 8-mm ePTFE and 12-mm woven graft (Fig 3). Postdilation was performed using a 10-mm balloon.

**Fig 4:**
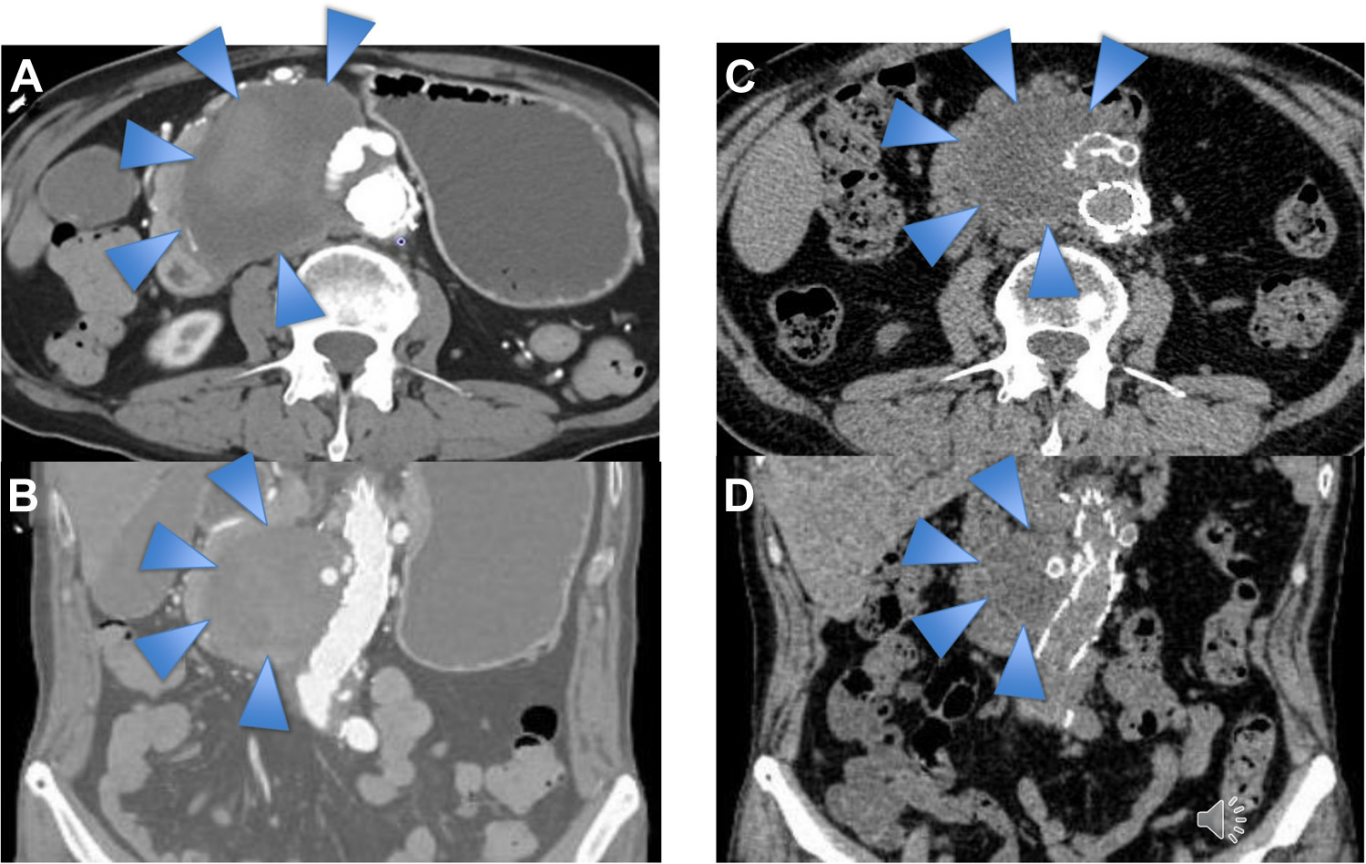
Computed tomography images at admission and 3 months after endovascular repair. **A,** Axial view at admission. **B,** Coronal view at admission. **C,** Axial view at 3 months after endovascular repair. **D,** Coronal view at 3 months after endovascular repair. The obtained images showed that the seroma (*blue arrows*) had decreased in size from 95 × 60 to 45 × 30 mm within 3 months after endovascular repair.

The postoperative course was uneventful. Soon after surgery, the daily drainage from the inserted nasogastric tube of 500 to 1100 mL had dramatically decreased to 0 mL, and the duodenal obstruction had resolved. The patient was carefully observed and discharged on day 15 after surgery. At the 3-month follow-up examination, the seroma size had decreased to 40 × 35 mm (Fig 4). The patient provided written informed consent for the report of his case details and imaging studies.

## Discussion

Serous ultrafiltrate extravasation through the graft wall can eventually result in a PS. Effective treatments include replacement of the affected graft, external surface sealing by application of fibrin glue components, and wrapping the graft with the saphenous vein, providing coverage for the pores outside the graft.^2^^,^^4^^,^^5^ However, those will require repeat laparotomy, an invasive procedure. Endovascular repair methods that do not require repeat laparotomy and are minimally invasive for covering pores inside the graft have been recently developed, with seven cases that had used AneuRx (Medtronic, Minneapolis, MN) and Excluder and Viabahn (W.L. Gore & Associates) devices reported^2^^,^^6^, ^7^, ^8^, ^9^ (Table). In those cases, repeat laparotomy was considered difficult because of the elderly age of the patient or a history of multiple laparotomies. The symptoms due to gastrointestinal compression caused by PS were improved by endovascular repair alone in each patient within 1 or 2 days (Table). Moreover, none of those studies, which had had a mean follow-up period of 19 months, had reported any recurrence or additional intervention needed, including drainage.

**Table:** Reported cases of endovascular repair for perigraft seroma (PS)^a^

Investigator	Age, years; sex	Diagnosis; initial procedure	Symptoms	Postoperative time to PS detection	Primary graft material	Endovascular graft	Endovascular graft site	Additional procedure for PS after endovascular repair	Time to remission after endovascular repair	Follow-up, months
Salameh et al, 2008	75; M	AAA; GR	Back pain	5 Years	ePTFE	AneuRx^b^	Replaced Y graft	No	NR	24
	75; M	AAA; GR	Abdominal and back pain	11 Years	ePTFE	AneuRx^b^	Replaced Y graft	No	NR	18
Sangawa et al,^1^ 2013	81; F	AAA; GR	Vomiting	24 Months	ePTFE	Excluder^c^	Replaced Y graft	No	2 Days	24
Lachat et al,^7^ 2013	57; M	TAAA; visceral debranching TEVAR	Abdominal discomfort	28 Months	ePTFE	Viabahn^c^	Bypass graft to SMA; bypass graft to CA	No	NR	18
	64; F	TAAA; visceral debranching TEVAR	NR	NR	ePTFE	Viabahn^c^	Bypass graft to SMA; bypass graft to CA	No	NR	10
Landis et al,^8^ 2018	78; M	AAA; GR	Abdominal and back pain	6 Years	ePTFE	NR	Replaced Y graft	No	NR	NR
Ono et al,^9^ 2018	86; M	AAA; GR	Abdominal distension and discomfort	6 Years	NR	Excluder^c^	Replaced Y graft	No	NR	24
Present patient, 2022	48; M	TAAA; visceral debranching TEVAR	Vomiting	8 Years	ePTFE	Viabahn VBX^c^	Bypass graft to right RA	No	1 Day	3

*AAA,* Abdominal aortic aneurysm; *CA,* celiac artery; *ePTFE,* expanded polytetrafluoroethylene; *GR,* graft replacement; *NR,* not reported; *RA,* renal artery; *SMA,* superior mesenteric artery; *TAAA,* thoracoabdominal aortic aneurysm; *TEVAR,* thoracic endovascular aortic repair.

a Details from a review of seven reported cases of endovascular repair of PS, including our patient; no patient had developed recurrence of the PS after surgery.

b Medtronic (Minneapolis, MN).

c W.L. Gore & Associates (Flagstaff, AZ).

Endovascular repair of a PS is a local procedure, with only the stent graft site receiving treatment; thus, identification of the affected graft and information regarding the PS site and graft material are necessary. It has been reported that a seroma will generally form around an affected graft, with ePTFE grafts more frequently involved than Dacron grafts.^10^ In the present patient, the PS was localized around the right RA ePTFE graft and, thus, was suspected to be the causative factor. Because of the site and graft material, stent relining for that alone was considered achievable. Also, Lachat et al^7^ reported two cases of endovascular repair for PS after visceral bypass surgery, for which stent relining was performed only for the affected graft because of the PS location and graft material. For cases of PS after visceral bypass surgery, the affected graft can be determined by the PS location and graft material composition, allowing for use of a minimally invasive intervention method.

The pathogenesis of PS formation remains unclear, although graft porosity, inadequate tissue incorporation, and improper manipulation before implantation are possible factors.^11^ For the prevention of PS, gentle manipulation of the graft is required. However, identification in a clinical setting is difficult, and the condition has remained a diagnosis of exclusion. Measurement of CT attenuation of perigraft fluid has been considered to have diagnostic value, although various criteria for PS shown by CT attenuation have been reported.^10^, ^11^, ^12^ In two large case series of PS development after aortic reconstruction, the interval from the initial procedure to seroma detection had a wide range from 3 to 156 months^10^^,^^11^ (Table).

Exclusion of possible graft infection is crucial for the correct diagnosis of PS. Physical findings, inflammatory markers (eg, C-reactive protein, white blood cell count), and negative CT findings related to a graft infection, such as gas formation or perigraft soft tissue abnormalities, are helpful. ^18^F-fluorodeoxyglucose positron emission tomography/CT can also help to exclude the possibility of graft infection.^13^ If a safe procedure is possible, aspiration of perigraft fluid for microbiologic analysis is an important option for the final diagnosis and should be considered.

## Conclusions

We have reported the successful endovascular repair of a PS that had developed around a visceral bypass graft after a hybrid thoracoabdominal aortic repair procedure. Relining of the visceral bypass graft with a covered stent resulted in immediate recovery of the duodenal obstruction as a seroma-related complication, indicating that endovascular repair can be an effective treatment option for PSs.
